# An Examination of the Ethnicity-Specific Prevalence of and Factors Associated with Substance Use and Misuse: Cross-Sectional Analysis of Croatian and Bosniak Adolescents in Bosnia and Herzegovina

**DOI:** 10.3390/ijerph13100968

**Published:** 2016-09-29

**Authors:** Dusko Bjelica, Kemal Idrizovic, Stevo Popovic, Nedim Sisic, Damir Sekulic, Ljerka Ostojic, Miodrag Spasic, Natasa Zenic

**Affiliations:** 1 Faculty for Sport and Physical Education, University of Podgorica, Niksic 81400, Montenegro; sportmont@t-com.me (D.B.); kemo@t-com.me (K.I.); stevop@ac.me (S.P.); 2 Faculty of Kinesiology, University of Split, Split 21000, Croatia; nedimsisic@hotmail.com (N.S.); damir.sekulic@kifst.hr (D.S.); ljerka.ostojic@sve-mo.ba (L.O.); miodrag.spasic@kifst.hr (M.S.); 3 University of Zenica, Zenica 72000, Bosnia and Herzegovina; 4 University Department of Health Care Studies, University of Split, Split 21000, Croatia; 5 Faculty of Medicine, University of Mostar, Mostar 88000, Bosnia and Herzegovina; 6 Academy of Medical Sciences, Sarajevo 71000, Bosnia and Herzegovina

**Keywords:** tobacco, alcohol, drugs, ethnic differences, logistic regression

## Abstract

Substance use and misuse (SUM) in adolescence is a significant public health problem and the extent to which adolescents exhibit SUM behaviors differs across ethnicity. This study aimed to explore the ethnicity-specific and gender-specific associations among sports factors, familial factors, and personal satisfaction with physical appearance (i.e., covariates) and SUM in a sample of adolescents from Federation of Bosnia and Herzegovina. In this cross-sectional study the participants were 1742 adolescents (17–18 years of age) from Bosnia and Herzegovina who were in their last year of high school education (high school seniors). The sample comprised 772 Croatian (558 females) and 970 Bosniak (485 females) adolescents. Variables were collected using a previously developed and validated questionnaire that included questions on SUM (alcohol drinking, cigarette smoking, and consumption of other drugs), sport factors, parental education, socioeconomic status, and satisfaction with physical appearance and body weight. The consumption of cigarettes remains high (37% of adolescents smoke cigarettes), with a higher prevalence among Croatians. Harmful drinking is also alarming (evidenced in 28.4% of adolescents). The consumption of illicit drugs remains low with 5.7% of adolescents who consume drugs, with a higher prevalence among Bosniaks. A higher likelihood of engaging in SUM is found among children who quit sports (for smoking and drinking), boys who perceive themselves to be good looking (for smoking), and girls who are not satisfied with their body weight (for smoking). Higher maternal education is systematically found to be associated with greater SUM in Bosniak girls. Information on the associations presented herein could be discretely disseminated as a part of regular school administrative functions. The results warrant future prospective studies that more precisely identify the causality among certain variables.

## 1. Introduction

Bosnia and Herzegovina is a country on the Balkan Peninsula, one of the former Yugoslav republics. During the last few years, studies have established a high prevalence of substance use and misuse (SUM) in the overall population, but the prevalence among adolescents is especially alarming [1,2]. In brief, daily smoking in Europe ranges mostly between 10%–20%, while 20% of adolescents in Bosnia and Herzegovina smoke on a daily basis [3,4]. Thus, Bosnia and Herzegovina, together with Hungary (18% daily smokers), Belgium (18%), and Austria (20%), is among the European countries with the highest prevalence of adolescent smoking [5,6]. Not surprisingly, high prevalence of smoking is recognized as one of the most important risk factors for high risk of morbidity and mortality related to cardiovascular diseases in the country [7,8]. The prevalence of 30% adolescents who reported harmful alcohol drinking (as measured by Alcohol Use Disorders Identification test (AUDIT)) is also disturbing [9,10]. More specifically, when evidenced by the same measuring tool (i.e., ten-item AUDIT scale), the higher rates of harmful alcohol drinking are evidenced only for Kosovar adolescents (35%) [11,12]. Meanwhile, when compared with the prevalence in other European countries, the consumption of drugs (opiates, prescription drugs, cannabinoids, etc.) in Bosnia and Herzegovina is low. In short, the average value for lifetime-ever use of illicit drugs in Europe is approximated at 15% to 20% [3]. Meanwhile, 7% of Bosnian and Herzegovinian adolescents reported lifetime use of illicit drugs, which is together with Montenegro (7.5%), and Norway (5%) the lowest prevalence of consumption in Europe [3].

The extent to which adolescents exhibit SUM behaviors differs across a number of sociocultural factors, including ethnicity [13,14,15,16]. The studies conducted thus far fairly consistently show ethnic differences in certain SUM templates, but there is an evident lack of investigations that examined the ethnicity-specific prevalence and correlates of SUM in European countries [17,18,19,20]. Also, in European studies authors mostly compared one-specific ethnic group to another heterogeneous group which included several different ethnicities [17,18]. For example, a Slovak study compared Roma and non-Roma adolescents, and reported lower prevalence of drunkenness among Roma adolescents [17], while a study from The Netherlands compared Dutch students with their ethnic-minority peers, and found lower prevalence of alcohol drinking among ethnic-minority groups [18]. Additionally, it is generally known the ethnic differences actually extrapolate the differences in socioeconomic status (SES) and scholastic status (i.e., differences in the availability of education, differences in the quality of the education system), which consequently result in different SUM templates between studied ethnicities [17,19,21].

It is rare to find a situation such as that in Bosnia and Herzegovina, where ethnic groups consistently speak the same language (i.e., there are some differences but they are “formal” and not “actual”), participate in equal scholastic systems (with the majority attending the same schools), participate in the same sport clubs and have similar sociocultural (i.e., ex-Yugoslav) backgrounds. More precisely, the ethnic differences among the ethnic groups in Bosnia and Herzegovina are represented only in inherited religious beliefs. Ethnic Croats are mostly Roman Catholic, ethnic Bosniaks are Muslim, and ethnic Serbs are Christian Orthodox. Knowing the certain religiously-inherited boundaries toward certain substances (e.g., alcohol with regard to Islam), and/or traditional perception of some SUM behaviors (i.e., tobacco smoking as a part of “eastern” and Arab culture), differences in SUM behaviors and factors associated to SUM among adolescents can be expected. Indeed, when observing the previous studies that investigated exclusively one ethnicity done on Croatian [1] and Bosniak adolescents [2,5] from Bosnia and Herzegovina, several conclusions can be made. First, Croatian boys consume alcohol to greater extent than Bosniaks (47% and 40%, respectively). Surprisingly, the alcohol drinking is higher in Bosniak (27%) than in Croatian girls (18%) of the same age. Next, there was substantial gender-difference in smoking (35% and 16% for Croatian boys and girls, respectively), while a very recent study which examined exclusively Bosniaks reported no significant difference between boys and girls in occurrence of daily smoking (30% and 32% for boys and girls, respectively) [5].

Investigations done so far frequently reported gender-specific relationships among different factors of influence on SUM [2,12,22]. For example, parental-variables (i.e., conflict with parents, lower parental monitoring) are indicated as risk factors for alcohol drinking in girls, but not in boys [2]. Furthermore, SES is oppositely related to alcohol drinking in adolescent boys and girls, with higher likelihood of drinking among girls of higher SES, and boys of lower SES [5]. Therefore, gender-specific analyses of the factors associated with SUM are suggested [2].

In their recent efforts to develop cost-effective and efficient preventive programs combating SUM, public health authorities and academicians from the region place special emphasis on the study of factors that may be related to SUM in adolescence [1,22]. Specifically, the period of adolescence is considered to be crucial in preventing SUM. Namely, adolescents who reach 21 years of age without smoking, drinking alcohol and consuming drugs are likely to never engage in these behaviors [12]. Therefore, it is particularly important to define any factor that could be directly and/or indirectly associated to SUM in this period of life. Studies reported several issues that should be specifically targeted in SUM prevention programs [5,12]. One of such factors is sport participation.

Sport provides numerous benefits to young people, and it is hypothesized that participation in sports could be protective against SUM [23]. There are two main theories on possible positive influence of sport participation on SUM in adolescence. First, the physical capacities necessary for sports are directly burden by the misuse of substances; therefore, athletes should avoid certain substances (i.e., cigarettes) because they alter physical performance and related sports achievement [1]. Second, sports promote social well-being and positively influence overall psychological status; therefore, adolescents who are involved in sports are (theoretically) less likely to initiate SUM [24]. However, the studies conducted thus far do not show consistent findings with regard to the association that may exist between sports participation and SUM in adolescence. While some authors reported a positive association between sports participation and SUM, others noted negative relationships and highlighted that athletic adolescents have a higher likelihood of engaging in certain types of SUM [24,25,26].

Although some factors are confirmed to be directly associated with higher levels of SUM (lower scholastic achievement, higher levels of conflict with parents), the applicability of these factors to the development of effective public health prevention programs is questionable. For example, establishing a causal pathway for associations between parental conflict and SUM and/or educational success/failure and SUM may be scientifically interesting but may not substantially add to our understanding of how to intervene. One can argue that this is because of the cross-sectional nature of the studies conducted thus far. However, even an attempt to assign a direction (i.e., causality) is not likely to inform the development of an intervention beyond simple knowledge that these factors co-occur. Therefore, future studies should focus on variables that could be effectively used in the development of preventive programs [2,5].

Consequently, the aim of this study was to explore the ethnicity-specific and gender-specific associations among sports factors, familial factors, and personal satisfaction with physical appearance (i.e., covariates) and SUM in a sample of adolescents from the Federation of Bosnia and Herzegovina. Additionally, we examined and compared the ethnicity-specific and gender-specific prevalence of SUM. In this study, we specifically investigated the problems among same-age ethnic Bosniak and Croatian adolescents from Bosnia and Herzegovina. Defining the ethnicity-specific prevalence and factors associated with SUM in different ethnic groups will help us to understand the SUM templates and to differentiate general vs. ethnicity-specific factors associated to consumption of different types of substances (i.e., cigarettes, alcohol and drugs). The leading hypotheses of this investigation were: (i) studied ethnicities will differ in SUM behavior; and (ii) gender-specific and ethnicity-specific relationships between studied covariates and SUM will be identified.

## 2. Materials and Methods

### 2.1. Participants

The participants in this study were 1742 adolescents (17–18 years of age) who were randomly selected by schools from three Cantons in Bosnia and Herzegovina. Originally, the sample included 1965 participants; however, for the purpose of this investigation, we observed only those who declared themselves to be ethnic Croats (*n* = 772; 558 females) and Bosniaks (*n* = 970; 485 females). All subjects were in their last year of high school education (i.e., 4th-year high school seniors). The sample was limited to this age group to be objectively comparable with studies conducted to date in the former Yugoslav territories [1,12,22]. Initially, 10 high schools for each Canton were selected by lottery, which resulted in 122 high-school senior classes and theoretical sample of 3676 children. We have decided to test all children from one school shift in each selected school (approximately 50% of the theoretical sample). Finally, testing was done only once, meaning that we tested children who were at school on a testing day. The testing was done on late November 2015.

### 2.2. Variables

Variables were collected by means of the Questionnaire of Substance Use (QSU), which was previously reported to be reliable and valid on the territory of former Yugoslavia [1,12,22]. In addition to collecting sociodemographic data (age, gender and ethnicity), the QSU asks participants questions about sports factors, familial variables, self-perceived physical appearance (i.e., covariates) and SUM (cigarette smoking, alcohol drinking and consumption of other drugs).

Sports factors consisted of questions asking subjects about their involvement in sports (answers included: never been involved, quit, currently involved) and sports competitive achievements (never competed/didn’t participate in sports, local rank competitions, National and International rank). Self-perceived physical appearance was measured by two questions, one question on appearance satisfaction and the other on weight perception, both answered on a four-point scale (very satisfied, satisfied, unsatisfied, very unsatisfied). Familial variables consisted of questions about paternal and maternal education levels, both answered on a three-point scale (elementary school, high school, college/university degree), and self-estimated SES (below average, average, above average).

SUM data included questions on cigarette smoking, alcohol consumption and consumption of other drugs. Cigarette smoking was assessed on a six-point scale including the following responses: “Never smoked”, “Quit”, “From time to time, but not daily”, “Less than 10 cigarettes daily”, “10–20 cigarettes daily”, and “More than a pack daily”. Participants were later classified into two groups: non-smokers (those who responded “Never smoked” and “Quit”) and smokers (the remaining four answers). Additionally, we have reported daily-smoking prevalence also (i.e., for those participants who responded “less than 10 cigarettes daily”, and higher). Alcohol consumption was measured using the AUDIT questionnaire, which contains 10 items with scores ranging from 0 to 4, resulting in a hypothetical minimum (0) to maximum (40) range. The results were later divided into “harmful drinking” (scores of 11 or above) and “non-harmful drinking” (scores below 11) [9,11]. Drug consumption was assessed by questions about the lifetime consumption of hashish, marijuana, cocaine, heroin, and most of the “party drugs” (e.g., speed, ecstasy, amphetamines, and others). A seven-point range of consumption was offered for each question (ranging from “never”, “once-twice”, to “10 times or more”). Those who declared that they consumed at least one type of drug more than “once-twice” and/or used two or more drugs “once-twice” were labeled as “drugs users” (“non-users” otherwise). The complete questionnaire used in this study is enclosed (Supplementary Materials).

### 2.3. Testing and Ethics

The survey was strictly anonymous, administered to groups of at least 15 respondents and contained multiple-choice answers. Each respondent received the questionnaire form and one envelope. After completing the survey, the subjects placed the questionnaire form in the envelope, sealed it and then placed it in the closed box. One week before study participants were informed of the study aims and procedure, written consent was obtained from their parents. The response rate was over 99%. The study fulfilled all ethical guidelines and received the approval of the ethical board of University of Split, Faculty of Kinesiology (EB: 2181-205-02-05-14-005; 11-Sep-2014). Additionally, the study was formally accepted by Cantonal Ministries of Education.

### 2.4. Statistics

Statistics included counts (frequencies) and percentages (for non-parametric variables), and means and standard deviations (for AUDIT scores). The differences between the two ethnicities were evidenced by calculating the Mann Whitney test (MW) and *t*-test for independent samples. The difference in the prevalence of SUM was compared by calculating the odds ratio (OR) with 95% confidence interval (95% CI). More precisely, we have compared users and non-users between genders (for total sample, and separately for Bosniaks and Croats), and between ethnicities (for total sample, and separately for males and females). Simple logistic regression with age as a confounding factor was applied to determine the association between the studied covariates and the SUM nominal criteria (see previous text for details on the classification of each SUM variable). Logistic regressions were ethnicity-stratified and gender-stratified. Statistica version 12.0 (Statsoft, Tulsa, OK, USA) was used for all calculations.

## 3. Results

**Figure 1:**
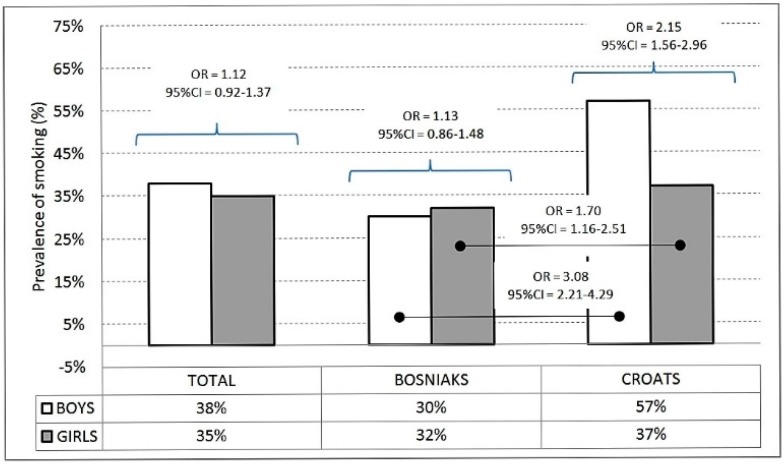
Prevalence of cigarette smoking with odds ratio between and within genders (OR: Odds Ratio; CI: Confidence Interval). Percentage of reported frequency for boys (the 100% being 214 and 485 for Croats and Bosniaks, respectively) and girls (the 100% being 558 and 485 for Croats and Bosniaks, respectively).

Of the adolescents, 37% smoke cigarettes, with a higher prevalence among boys for the total sample and among Croatian adolescents. When comparing the ethnicities, cigarette smoking is more prevalent among Croats for the total sample, boys and girls (Figure 1). The 30% of participants are identified as daily smokers (29% and 31% for Croatian and Bosniaks, respectively).

**Figure 2:**
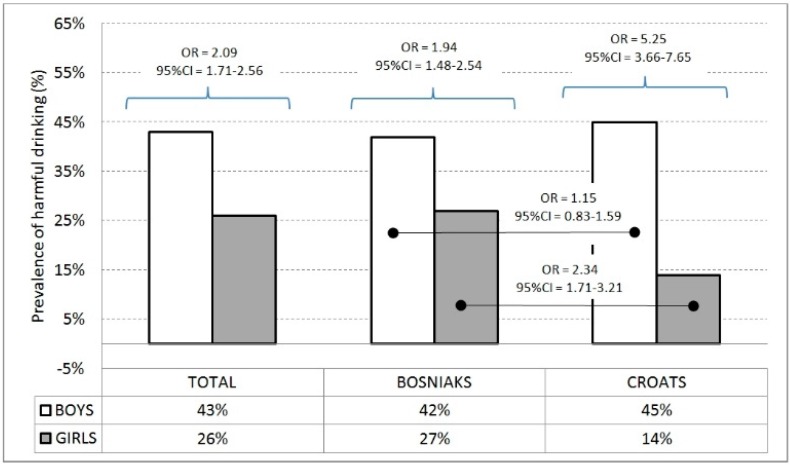
Prevalence of harmful alcohol drinking with odds ratio between and within genders. Percentage of reported frequency for boys (the 100% being 214 and 485 for Croats and Bosniaks, respectively) and girls (the 100% being 558 and 485 for Croats and Bosniaks, respectively).

Harmful drinking is evidenced in 28.4% of adolescents, with a higher prevalence among boys in the total sample and in both ethnic groups. Bosniak girls are more likely to engage in harmful drinking than their Croatian peers (Figure 2).

**Figure 3:**
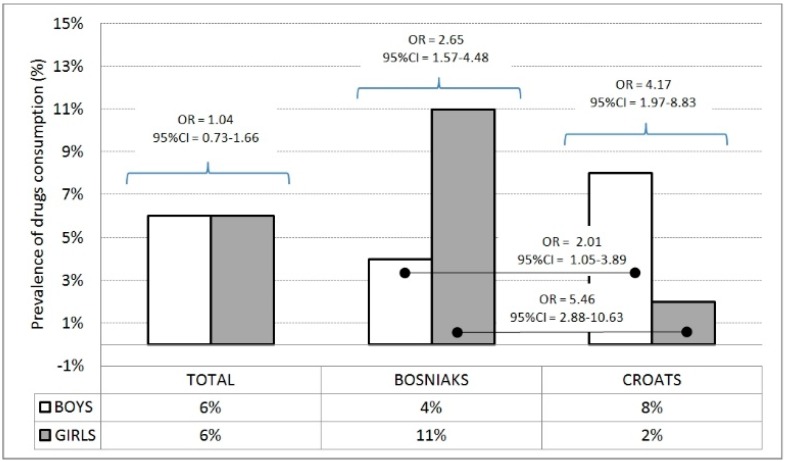
Prevalence of consumption of other drugs with odds ratio between and within genders. Percentage of reported frequency for boys (the 100% being 214 and 485 for Croats and Bosniaks, respectively) and girls (the 100% being 558 and 485 for Croats and Bosniaks, respectively).

Of the adolescents, 5.7% consume drugs. In the total sample and the sample of Bosniak adolescents, the prevalence of drug consumption is higher among girls. Croatian boys consume drugs more often than Croatian girls. The prevalence of drug consumption is higher among Bosniak girls than among Croatian girls, resulting in an overall higher prevalence of consumption of drugs among Bosniak adolescents than among their Croatian peers (Figure 3).

When the overall AUDIT scores of the two ethnic groups are compared, significant differences are found for the total sample (6.39 ± 6.90 and 8.79 ± 8.60 for Croatian and Bosniak adolescents, respectively, *t*-test = 6.27; *p* < 0.001) and for girls (4.79 ± 5.56 and 7.45 ± 8.33 for Croatian and Bosniak girls, respectively, *t*-test = 6.08; *p* < 0.001) but not for boys (10.48 ± 8.21 and 10.13 ± 8.67 for Croatian and Bosniak adolescents, respectively, *t*-test = 0.49; *p* = 0.62).

When Croatian and Bosniak boys are compared on the independent variables, Croatian boys report greater sports achievement (21% and 8% with National/International rank for Croats and Bosniaks, respectively; Mann Whitney Z-test (MWZ) = 2.48; *p* < 0.001), higher levels of maternal education (33% and 13% with College/University maternal educational level for Croats and Bosniaks, respectively; MWZ = 5.57; *p* < 0.001) and higher levels of satisfaction with their physical appearance (22% and 7% who are satisfied with own physical appearance among Croats and Bosniaks, respectively; MWZ = 4.89; *p* < 0.001).

Bosniak girls report lower levels of maternal education (32% and 24% with College/University degree for Croatian and Bosniak girls, respectively; MWZ = 6.01; *p* < 0.001) and paternal education (7% and 28% with Elementary school for Croatian and Bosniak girls, respectively; MWZ = 5.17; *p* < 0.001) than Croatian girls, while Croatian girls report less satisfaction with their weight than Bosniak girls (17% and 8% who are not satisfied with own physical appearance for Croatian and Bosniak girls, respectively; MWZ = 6.21; *p* < 0.001).

For Croatian girls, the higher likelihood of SUM is evidenced for those girls who once practiced sport and then quit (OR = 2.03; 95% CI = 1.35–3.08, *p* < 0.001; and OR = 2.37; 95% CI = 1.46–3.87, *p* < 0.001, for cigarette smoking and drugs consumption, respectively, with current sport participation as reference value (REF)), and those girls who achieved lower competitive success in sports (OR = 1.70; 95% CI = 1.09–2.64, *p* < 0.05; and OR = 2.54; 95% CI = 1.48–4.34, *p* < 0.001, for cigarette smoking and drugs consumption, respectively). Those girls who were never involved in sports are less likely to be smokers than those who are currently involved in sports (OR = 0.42; 95% CI = 0.23–0.89, *p* < 0.05). Harmful drinking is higher in girls who are not absolutely satisfied with own physical appearance (“satisfied”: OR = 4.12; 95% CI = 1.44–11.97, *p* < 0.001; “unsatisfied”: OR = 4.95; 95% CI = 1.48–16.56, *p* < 0.001; “very unsatisfied”: OR = 42.08; 95% CI = 10.15–174.22, *p* < 0001 (Table 1).

When calculated for Bosniak girls, logistic regression evidenced higher likelihood of drugs consumption in non-athletic girls (OR = 2.43; 95% CI = 1.17–5.05, *p* < 0.05) and those who quit sports (OR = 1.82; 95% CI = 1.17–2.82, *p* < 0.001). Smoking is more prevalent in those who achieved lower sport success than in those who were never involved in sports (OR = 2.11; 95% CI = 1.37–3.24, *p* < 0.001). The prevalence of harmful drinking is higher in those who practiced sports but achieved lower competitive result (OR = 5.39; 95% CI = 2.59–11.19, *p* < 0.001), harmful alcohol drinking is higher in those who achieved National/International result (OR = 5.39; 95% CI = 2.59–11.19, *p* < 0.001), while consumption of illicit drugs is more prevalent in athletes (National/International rank: OR = 2.57; 95% CI = 1.03–6.39, *p* < 0.05; low rank: OR = 2.49; 95% CI = 1.49–4.15, *p* < 0.01) than in those Bosniak girls who were never involved in sports. Those girls whose fathers had finished high school education have lower prevalence of harmful drinking (OR = 0.35; 95% CI = 0.20–0.61, *p* < 0.001). Similarly, lower tendency toward harmful drinking (high school: OR = 0.48; 95% CI = 0.27–0.86, *p* < 0.05; elementary school: OR = 0.30; 95% CI = 0.16–0.58, *p* < 0.001) and consumption of drugs (high school: OR = 0.13; 95% CI = 0.05–0.37, *p* < 0.001; elementary school: OR = 0.30; 95% CI = 0.04–0.39, *p* < 0.001) is found in those girls whose mothers were relatively less educated. Harmful drinking is less prevalent in those who are very unsatisfied (OR = 0.21; 95% CI = 0.05–0.97, *p* < 0.05), and unsatisfied (OR = 0.21; 95% CI = 0.06–0.72, *p* < 0.05) with own physical appearance. Also, those who are very unsatisfied with own physical appearance are less likely to consume drugs (OR = 0.38; 95% CI = 0.15–0.96, *p* < 0.05). The higher likelihood of cigarette smoking (OR = 1.36; 95% CI = 1.19–2.35, *p* < 0.001), and consumption of illicit drugs (OR = 2.46; 95% CI = 1.18–5.10, *p* < 0.01) is evidenced in those Bosniak girls who are very unsatisfied with their body weight (Table 2).

When compared to those currently involved in sports, smoking and harmful drinking are more prevalent in those Croatian boys who quit sports (smoking: OR = 2.47; 95% CI = 1.54–3.77, *p* < 0.001; drinking: OR = 2.14; 95% CI = 1.35–3.37, *p* < 0.001), while smoking and consumption of illicit drugs is lower in those who were never involved in sports (smoking: OR = 0.56; 95% CI = 0.34–0.92, *p* < 0.05; drugs: OR = 0.41; 95% CI = 0.20–0.85, *p* < 0.05). Those boys who achieved high sport success (i.e., National/International rank) are less likely to be smokers than those who were never involved in sports (OR = 0.42; 95% CI = 0.23–0.75, *p* < 0.001). Lower SES is associated with lower prevalence of illicit drugs consumption (OR = 0.05; 95% CI = 0.08–0.29, *p* < 0.001). Lower prevalence of smoking is evidenced in boys whose mothers finished elementary school (OR = 0.40; 95% CI = 0.16–0.99, *p* < 0.05). Those Croatian boys who declared relative dissatisfaction with own physical appearance are less likely to be smokers (OR = 0.25; 95% CI = 0.12–0.53, *p* < 0.001), while those very unsatisfied with own physical appearance are more likely to be engaged in harmful drinking (OR = 3.03; 95% CI = 1.48–6.28, *p* < 0.001) (Table 3).

The higher likelihood of smoking (OR = 1.81; 95% CI = 1.11–2.29, *p* < 0.01) and consumption of drugs (OR = 3.07; 95% CI = 1.62–5.85, *p* < 0.001) is evidenced for those Bosniak boys who quit sports, than in those who reported current involvement in sports. The consumption of drugs is more prevalent in those who were never involved in sports (OR = 2.07; 95% CI = 1.11–3.87, *p* < 0.05), and those who achieved lower competitive result (OR = 1.75; 95% CI = 1.03–2.96, *p* < 0.05). Self-reported “below-average” and “average” SES are connected with higher likelihood of smoking (below-average SES: OR = 5.66; 95% CI = 1.05–30.51, *p* < 0.05; average SES: OR = 5.28; 95% CI = 1.59–17.49, *p* < 0.001). The cigarette smoking (OR = 2.08; 95% CI = 1.32–3.27, *p* < 0.001) and harmful drinking (OR = 1.74; 95% CI = 1.17–2.59, *p* < 0.001) are higher in boys who are moderately satisfied-, in comparison to those who are highly satisfied with own physical appearance. Finally, harmful drinking behavior is more frequent for Bosniak boys who are “very unsatisfied” (OR = 2.38; 95% CI = 1.09–5.20, *p* < 0.05) and “unsatisfied” with own body weight (OR = 1.77; 95% CI = 1.06–2.95, *p* < 0.05) (Table 4).

## 4. Discussion

This is the first investigation to systematically examine the ethnicity-specific prevalence of SUM and factors associated with SUM among adolescents in the territory of former Yugoslavia. Although the presented results allow a broad discussion, we will focus solely on the most important findings with regard to the study aims. First, there is an evident ethnicity-specific prevalence of SUM in the studied sample of adolescents. Second, sports factors and satisfaction with physical appearance are related to SUM among Croatian and Bosniak adolescents. Finally, parental education (for girls) and SES (for boys), is a strong factor of influence on SUM among Bosniak adolescents.

### 4.1. Prevalence of and Difference in SUM between the Two Ethnicities

The studies conducted thus far reported a high prevalence of SUM among adolescents from Bosnia and Herzegovina [1,9]. Showing the overall prevalence of 37% smokers and 28% adolescents who were engaged in harmful alcohol drinking, our results support previous findings. There are several explanations for such disturbing SUM figures. First, the prices of alcohol and tobacco products are relatively low [1]. Next, on the territory of Bosnia and Herzegovina, smoking and alcohol drinking are socially acceptable behaviors. These behaviors are additionally fostered by the more than 300-year-old tradition of tobacco growing as well as wine production in the Mediterranean part of the country (i.e., Herzegovina-Neretva Canton) [9]. Finally, recent wars resulted in long-lasting post-war trauma; thus, the country’s authorities focused on complex post-war political issues.

Croatian adolescents consume more cigarettes, as evidenced in the total sample and when the analysis was stratified by gender. The previous studies that studied a similar sample in 2011 showed a very similar prevalence of smoking [1]. It has been suggested that the tradition of tobacco farming (growing) in the region of Bosnia and Herzegovina where Croatians primarily reside (i.e., Herzegovina Neretva Canton) is one of the most important reasons for the overall social acceptance of tobacco smoking, resulting in the high prevalence of adolescent smoking [1]. The current authors are familiar with another issue that was not specifically studied herein but deserves attention.

On several occasions, Bosniak subjects noted that they “don’t smoke cigarettes but consume narghile” (i.e., they wrote this information on the questionnaire without prompting). Because the questionnaires were unsealed following next day (for details on testing see previous text on Testing and Ethics), we were not able to include narghile on the form and to explore this problem more precisely. However, given that some of the authors reside in Bosnia and Herzegovina, we may assume based on observation that narghile smoking is practically unknown in the Croatian ethnic group. Therefore, it seems reasonable to assume that narghile smoking to some extent “decreased” the prevalence of cigarette smoking among Bosniak adolescents.

The prevalence of harmful drinking is higher among Bosniak adolescents, but this result is exclusively related to the difference between Croatian and Bosniak girls (i.e., no significant ethnic difference is found for boys). At first glance, this is an unexpected finding because of the known boundaries regarding alcohol drinking of Islamic religiousness [27]. However, we must note that a recent study conducted in other parts of former Yugoslavia (i.e., Kosovo) also reported a high prevalence of alcohol consumption among religiously Muslim adolescents, and especially among females [11]. This result is mostly explained by the relatively “liberal” type of Islamic culture in the whole territory of former Yugoslavia. Additionally, it is augmented by the fact that Muslim girls frequently present their “liberal tendencies” through habits that are traditionally not connected to their ethnicity and religion [2]. Regardless of the nature and background of alcohol consumption, the prevalence is disturbing and warrants urgent intervention.

While differences in drug consumption are gender-specific (i.e., Bosniak girls consume more drugs than their Croatian peers, while the figure is the opposite for boys), the overall prevalence of drug consumption is somewhat higher among Bosniaks. However, drug consumption is mostly related to cannabis and sedatives. In comparison with the figures in other European countries, these figures are not disturbing [3].

### 4.2. Sports Factors and Satisfaction with Physical Appearance and Their Association with SUM

The associations that exist between sports participation (physical exercise, training, etc.) and SUM in adolescence are frequently investigated [1,22,24]. This is one of the first studies that investigated sports variables as ethnicity-specific factors that influence SUM in adolescence. This issue is additionally interesting because the sociocultural background and overall importance of sports in Bosnia and Herzegovina are similar across both studied ethnicities. This is mostly because the two ethnicities share a similar social environment and the same scholastic system (see Introduction for details). Therefore, it is not surprising that the association between sports and SUM is similar across the two studied ethnicities. In general, the adolescents who previously practiced sports but quit (i.e., they are not actively involved in sports at the moment of testing) are at particular risk for SUM.

Our findings of a high prevalence of SUM among adolescents who discontinued sports support the recent literature that suggested that “changes in sports participation” is a factor potentially associated to SUM in adolescence [24]. There are two probable explanations for such findings. First, it is possible that SUM altered the physical capacities of those athletes who misused substances, resulting in poor athletic performance, frustration and quitting sports. Second, there is a possibility that some children stopped practicing sports (first) and then started to misuse substances while attempting to establish a social connection with their “non-sportive” peers. However, for a more profound analysis of these relationships, a longitudinal analysis is needed.

Weight perception and appearance satisfaction are known to be important health-related issues in adolescence, and authors repeatedly indicated the importance of these issues when evaluating potential factors associated with SUM in this age group [28,29,30]. Surprisingly, studies directly investigating these associations are scarce. The fact that smoking is more prevalent among the girls who are more satisfied with their appearance is highly specific, and we will discuss this result in relation to the results of studies that investigated this issue in other contexts. In brief, cigarette smoking increases basal metabolism and suppresses appetite. Therefore, smoking is common in occupations in which a lean and thin body is necessary for overall success (i.e., ballet, fashion modeling) [31,32]. This perception is probably reflected to the adolescent population as well [29]. Interestingly, body weight perception and satisfaction with overall physical appearance are oppositely related to harmful alcohol drinking in Bosniak girls (those who are not satisfied with appearance/body weight are less likely to consume alcohol) and Croatian girls (consumption is higher in those less satisfied with physical appearance). Since this is one of the first studies that investigated this issue, for detailed and elaborated conclusions, a more detailed qualitative analysis of adolescents’ motives and attitudes is needed.

### 4.3. Familial Factors and Their Association with SUM

A growing body of evidence shows that familial variables influence SUM among children. However, the results of the studies that examined these associations are not consistent. While some authors presented positive associations (i.e., lower prevalence of SUM among children whose parents were better educated) [33], others reported the opposite findings [34], and/or differential influence of paternal and maternal education on their children’s SUM [35]. Higher levels of maternal education increase the likelihood that Bosniak girls engage in SUM. We are fairly convinced that this association is directly modulated by lower levels of traditionalism in families in which females (i.e., mothers) are better educated [36]. The girls from “more liberal” families are more likely to consume substances [37]. Logically, and knowing the overall perception of alcohol drinking in Islamic religions, this is mostly evident for alcohol drinking. However, the fact that the association between maternal education and SUM is evidenced only for Bosniak girls deserves special attention. A possible explanation for this result is provided below.

After the devastating wars that occurred in the early 1990s, republics of former Yugoslavia faced an increase in traditionalism. Among ethnic Bosniaks, religious authorities and politicians frequently emphasize their oriental (i.e., religiously Muslim) tradition. This is additionally aggravated by the simple fact that Bosnia and Herzegovina is Bosniaks’ (only) “national” country. Meanwhile, ethnic Croats from Bosnia and Herzegovina are strongly oriented toward the Republic of Croatia because of education, business, familial relationships, or (at least) tourism. Consequently, ethnic Croats are more likely to be influenced by the tradition of the European Union and, therefore, by more liberal tendencies. To some extent this probably explains the lesser SUM among Bosniak girls whose mothers are less educated.

For Bosniak adolescents, lower SES is associated with a higher prevalence of smoking, but the opposite association is evident for alcohol drinking and consumption of drugs (exclusively in girls). These findings are generally consistent with previous studies [5]. Namely, smoking is allowed in public, and buying cigarettes is not a financial burden because of the low prices. By contrast, girls are permitted to drink alcohol only in clubs and bars, where alcohol is relatively expensive. Thus, girls with a better financial background have a higher likelihood of drinking.

### 4.4. Limitations

The main limitation of the investigation comes from the fact that the study is based on self-reporting. However, we tested the children at the end of their mandatory education which consequently decreased the possibility that they did not respond honestly. Also, the study is retrospective. For that reason we are unable to define the causality between variables. Since we have observed three criteria (i.e., smoking, drinking and consumption of illicit drugs), large set of covariates, and four subsamples of participants, we have calculated univariate analyses only, which almost certainly limited the possibility of the more profound discussion of the observed associations. Finally, the study is done in a country where smoking and alcohol drinking are generally socially acceptable. Therefore, the generalizability of the findings is limited. However, since this is probably the first study that has investigated the problem of ethnicity-specific prevalence of substance use and misuse in adolescents from the wider region (i.e., territory of former Yugoslavia), we believe that it will contributes to the body of knowledge on this field.

## 5. Conclusions

While the studied adolescents do not differ in main economic and/or social factors (availability of education, inclusion in sports, etc.), the identified differences in (i) SUM prevalence and (ii) factors associated with SUM between the ethnicities are particularly important. A higher level of maternal education is systematically found to be associated with greater SUM among Bosniak girls. This result is almost certainly related to the more “liberal” structure in families with highly educated females, who probably allow girls to engage in SUM. The high risk of SUM is evidenced in those children who quit sports. The underlying mechanisms of this association are not directly investigated in this study, and this issue should be more precisely explored in future research. The current study confirmed previous findings on (i) the lack of an association between SES and smoking (cigarettes are relatively cheap) and (ii) the higher likelihood of harmful alcohol drinking among girls with a better financial background (girls’ drinking behavior is socially acceptable only in clubs and bars, where alcohol is relatively expensive). The information on the association presented herein could be discretely disseminated as a part of regular school administrative functions. However, this information should be presented in such a way that parents are directly informed of a (potential) problem but are not labeled as “responsible”.

## Figures and Tables

**Table 1:** Logistic regression calculations for Croatian girls.

COVARIATES	Cigarettes	Alcohol	Drugs
	OR (95% CI)	OR (95% CI)	OR (95% CI)
SPORT PARTICIPATION			
Never	0.42 (0.23–0.89) *	1.67 (0.77–3.59)	1.01 (0.56–1.83)
Quit	2.03 (1.35–3.08) ***	1.48 (0.84–2.64)	2.37 (1.46–3.87) ***
Participating	REF	REF	REF
SPORT ACHIEVEMENT			
Not involved/not competed	REF	REF	REF
Low rank	1.70 (1.09–2.64) *	0.92 (0.48–1.76)	2.54 (1.48–4.34) ***
National/International rank	0.41 (0.16–1.04)	0.89 (0.29–2.63)	0.95 (0.45–2.02)
SOCIOECONOMIC STATUS			
Below average	1.01 (0.25–1.69)	0.55 (0.01–1.99)	0.96 (0.55–4.12)
Average	0.88 (0.56–2.58)	0.69 (0.11–5.24)	0.84 (0.21–8.69)
Above average	REF	REF	REF
PATERNAL EDUCATION			
Elementary school	0.94 (0.33–2.74)	0.75 (0.15–3.73)	3.21 (0.68–15.16)
High school	0.84 (0.51–1.39)	0.91 (0.46–1.84)	0.92 (0.54–1.56)
College/University	REF	REF	REF
MATERNAL EDUCATION			
Elementary school	1.38 (0.54–3.51)	0.72 (0.28–1.83)	0.55 (0.22–1.42)
High school	1.82 (0.94–3.52)	0.51 (0.16–1.59)	0.99 (0.51–1.93)
College/University	REF	REF	REF
APPEARANCE SATISFACTION			
Very unsatisfied	2.62 (0.90–7.64)	42.08 (10.15–174.22) ***	1.7 (0.51–5.65)
Unsatisfied	1.48 (0.75–2.90)	4.95 (1.48–16.56) ***	0.87 (0.46–1.68)
Satisfied	1.80 * (1.12–2.92)	4.12 (1.44–11.97) ***	1.36 (0.85–2.18)
Very satisfied	REF	REF	REF
WEIGHT SATISFACTION			
Very unsatisfied	2.23 (1.13–4.41) *	6.25 (2.41–16.26) ***	2.45 (1.08–5.57) *
Unsatisfied	1.67 (1.05–2.69) *	7.24 (3.43–15.32) ***	1.02 (0.64–1.62)
Satisfied	1.75 (1.10–2.79) *	2.43 * (1.04–5.64)	2.08 (1.25–3.45)
Very satisfied	REF	REF	REF

* *p* < 0.05; *** *p* < 0.001; REF: reference value.

**Table 2:** Logistic regression calculations for Bosniak girls.

COVARIATES	Cigarettes	Alcohol	Drugs
	OR (95% CI)	OR (95% CI)	OR (95% CI)
SPORT PARTICIPATION			
Never	1.20 (0.66–2.21)	0.81 (0.41–1.64)	2.43 (1.17–5.05) *
Quit	1.13 (0.75–1.69)	1.47 (0.95–2.25)	1.82 (1.17–2.82) ***
Participating	REF	REF	REF
SPORT ACHIEVEMENT			
Not involved/not competed	REF	REF	REF
Low rank	2.11 (1.37–3.24) ***	1.42 (0.88–2.23)	2.49 (1.49–4.15) ***
National/International rank	0.87 (0.40–1.92)	5.39 (2.59–11.19) ***	2.57 (1.03–6.39) *
SOCIOECONOMIC STATUS			
Below average	0.51 (0.00–17.64)	0.99 (0.41–2.11)	1.11 (0.21–1.88)
Average	0.57 (0.01–12.11)	1.02 (0.71–1.44)	1.03 (0.37–2.01)
Above average	REF	REF	REF
PATERNAL EDUCATION			
Elementary school	0.48 (0.19–1.17)	0.45 (0.19–1.07)	0.36 (0.15–0.89)
High school	0.75 (0.41–1.26)	0.35 (0.20–0.61) ***	0.53 (0.27–1.03)
College/University	REF	REF	REF
MATERNAL EDUCATION			
Elementary school	0.76 (0.40–1.47)	0.30 (0.16–0.58) ***	0.13 (0.04–0.39) ***
High school	1.06 (0.58–1.92)	0.48 (0.27–0.86) *	0.13 (0.05–0.37) ***
College/University	REF	REF	REF
APPEARANCE SATISFACTION			
Very unsatisfied	1.67 (0.67–4.18)	0.21 (0.05–0.97) *	0.38 (0.15–0.96) *
Unsatisfied	0.62 (0.26–1.48)	0.21 (0.06–0.72) *	2.22 (0.79–6.19)
Satisfied	0.95 (0.62–1.48)	0.86 (0.54–1.35)	0.99 (0.62–1.58)
Very satisfied	REF	REF	REF
WEIGHT SATISFACTION			
Very unsatisfied	1.36 (1.19–2.35) ***	0.96 (0.55–1.69)	2.46 (1.18–5.10) **
Unsatisfied	0.61 (0.37–1.02)	0.37 (0.21–0.66) **	0.65 (0.39–1.07)
Satisfied	0.69 (0.49–1.16)	0.61 (0.35–1.05)	0.93 (0.54–1.59)
Very satisfied	REF	REF	REF

* *p* < 0.05; ** *p* < 0.01; *** *p* < 0.001; REF: reference value.

**Table 3:** Logistic regression calculations for Croatian boys.

COVARIATES	Cigarettes	Alcohol	Drugs
	OR (95% CI)	OR (95% CI)	OR (95% CI)
SPORT PARTICIPATION			
Never	0.56 (0.34–0.92) *	1.22 (0.74–2.02)	0.41 (0.20–0.85) *
Quit	2.47 (1.54–3.77) ***	2.14 (1.35–3.37) ***	1.44 (0.64–3.22)
Participating	REF	REF	REF
SPORT ACHIEVEMENT			
Not involved/not competed	REF	REF	REF
Low rank	0.81 (0.52–1.28)	1.33 (0.84–2.05)	1.66 (0.85–3.29)
National/International rank	0.42 (0.23–0.75) ***	1.02 (0.57–1.82)	1.89 (0.79–4.95)
SOCIOECONOMIC STATUS			
Below average	0.42 (0.11–1.59)	1.44 (0.40–5.21)	0.05 (0.08–0.29) ***
Average	1.09 (0.59–2.05)	1.2 (0.64–2.26)	0.35 (0.08–1.51)
Above average	REF	REF	REF
PATERNAL EDUCATION			
Elementary school	1.14 (0.47–2.47)	0.69 (0.26–1.81)	0.74 (0.24–2.27)
High school	1.38 (0.80–2.41)	1.82 (0.97–3.23)	1.52 (0.69–3.32)
College/University	REF	REF	REF
MATERNAL EDUCATION			
Elementary school	0.40 (0.16–0.99) *	0.59 (0.22–1.59)	0.41 (0.17–1.31)
High school	0.87 (0.47–1.63)	1.8 (0.96–3.39)	1.08 (0.42–2.77)
College/University	REF	REF	REF
APPEARANCE SATISFACTION			
Very unsatisfied	0.96 (0.47–1.94)	3.03 (1.48–6.28) ***	0.48 (0.17–1.29)
Unsatisfied	0.25 (0.12–0.53) ***	1.62 (0.81–3.21)	1.1 (0.32–3.69)
Satisfied	0.85 (0.53–1.37)	1.21 (0.76–1.94)	0.65 (0.30–1.38)
Very satisfied	REF	REF	REF
WEIGHT SATISFACTION			
Very unsatisfied	0.71 (0.36–1.39)	1.41 (0.72–2.76)	0.76 (0.30–1.98)
Unsatisfied	1.35 (0.76–2.42)	1.59 (0.92–2.78)	2.01 (0.68–5.69)
Satisfied	0.65 (0.39–1.07)	1.29 (0.78–2.10)	0.71 (0.35–1.43)
Very satisfied	REF	REF	REF

* *p* < 0.05; *** *p* < 0.001; REF: reference value.

**Table 4:** Logistic regression calculations for Bosniak boys.

COVARIATES	Cigarettes	Alcohol	Drugs
	OR (95% CI)	OR (95% CI)	OR (95% CI)
SPORT PARTICIPATION			
Never	0.92 (0.54–1.58)	0.99 (0.62–1.59)	2.07 (1.11–3.87) *
Quit	1.81 (1.11–2.29) **	1.37 (0.87–2.15)	3.07 (1.62–5.85) ***
Participating	REF	REF	REF
SPORT ACHIEVEMENT			
Not involved/not competed	REF	REF	REF
Low rank	1.51 (0.98–2.31)	1.45 (0.98–2.16)	1.75 (1.03–2.96) *
National/International rank	0.73 (0.28–1.91)	0.86 (0.38–1.96)	3.39 (0.77–14.92)
SOCIOECONOMIC STATUS			
Below average	5.66 (1.05–30.51) *	2.62 (0.67–10.28)	0.96 (0.17–5.58)
Average	5.28 (1.59–17.49) ***	0.92 (0.46–1.82)	1.27 (0.51–3.18)
Above average	REF	REF	REF
PATERNAL EDUCATION			
Elementary school	2.53 (0.85–7.52)	1.38 (0.47–4.08)	2.91 (0.34–24.47)
High school	0.84 (0.47–1.51)	0.61 (0.36–1.04)	1.12 (0.54–2.35)
College/University	REF	REF	REF
MATERNAL EDUCATION			
Elementary school	1.24 (0.56–2.73)	0.47 (0.22–1.03)	0.26 (0.06–1.14)
High school	0.88 (0.43–1.83)	0.47 (0.24–1.11)	0.36 (0.08–1.56)
College/University	REF	REF	REF
APPEARANCE SATISFACTION			
Very unsatisfied	2.15 (0.59–7.78)	1.05 (0.29–3.75)	0.28 (0.08–1.02)
Unsatisfied	1.25 (0.49–3.19)	0.61 (0.25–1.53)	0.47 (0.18–1.24)
Satisfied	2.08 (1.32–3.27) ***	1.74 (1.17–2.59) ***	1.21 (0.68–2.16)
Very satisfied	REF	REF	REF
WEIGHT SATISFACTION			
Very unsatisfied	1.39 (0.62–3.15)	2.38 (1.09–5.20) *	2.01 (0.45–8.81)
Unsatisfied	1.11 (0.63–1.94)	1.77 (1.06–2.95) *	0.74 (0.37–1.49)
Satisfied	1.48 (0.92–2.38)	1.33 (0.84–2.09)	0.86 (0.45–1.64)
Very satisfied	REF	REF	REF

* *p* < 0.05; ** *p* < 0.01; *** *p* < 0.001; REF: reference value.

## Supplementary Materials

The supplementary materials are available online at www.mdpi.com/1660-4601/13/10/968/s1.
