# Supplementary material for: An Examination of the Ethnicity-Specific Prevalence of and Factors Associated with Substance Use and Misuse: Cross-Sectional Analysis of Croatian and Bosniak Adolescents in Bosnia and Herzegovina
Source: Int J Environ Res Public Health. 2016 Sep 29;13(10):968. doi: 10.3390/ijerph13100968 (PMC5086707; doi:10.3390/ijerph13100968)
Supplement: Supplementary file 1 [file ijerph-13-00968-s001.pdf]

Supplementary Materials: An Examination of the Ethnicity-Specific Prevalence of and Factors Associated with Substance Use and Misuse: Cross-Sectional Analysis of Croatian and Bosniak Adolescents in Bosnia and Herzegovina

Dusko Bjelica, Kemal Idrizovic, Stevo Popovic, Nedim Sisic, Damir Sekulic, Ljerka Ostojic, Miodrag Spasic and Natasa Zenic

"Questionnaire of Substance Use" used in Investigation on ethnicity-specific prevalence of substance use and misuse in Bosnia and Herzegovina

1. **Age:**
2. **Gender (please encircle):**

    Male Female

3. **Ethnicity (please encircle and/or write additional if necessary):**

    Bosniak Croatian Serbian Other:

4. **Participation (involvement) in sport:**
    a. Never been involved in sport
    b. I have been involved, but quit
    c. Currently involved
5. **Sports competitive achievement—result (please encircle the highest result)**
    a. Never competed/didn't participate in sports
    b. Local rank competitions
    c. National/International level competitions
6. **How satisfied are you with your physical appearance**
    a. Very unsatisfied
    b. Unsatisfied
    c. Satisfied
    d. Very satisfied
7. **How satisfied are you with your body weight**
    a. Very unsatisfied
    b. Unsatisfied
    c. Satisfied
    d. Very satisfied
8. **Maternal education**
    a. Elementary school
    b. High school
    c. College/University degree
9. **Paternal education**
    a. Elementary school
    b. High school
    c. College/University degree

10. Do you smoke cigarettes?

a. Never smoked
b. Quit
c. From time to time, but not daily
d. Daily, less than 10 cigarettes
e. 10–20 cigarettes daily
f. More than a pack daily

11. Please read the questions on the left, and encircle the correct answer on the right. After answering the first question please read the note at the bottom of the table.

1. How often do you have a drink containing alcohol?	Never	Monthly or less	2–4 times a month	2–3 times a week	4 or more times a week
2. How many standard drinks containing alcohol do you have on a typical day when drinking?	1 or 2	3 or 4	5 or 6	7 to 9	10 or more
3. How often do you have six or more drinks on one occasion?	Never	Less than monthly	Monthly	Weekly	Daily or almost daily
4. During the past year, how often have you found that you were not able to stop drinking once you had started?	Never	Less than monthly	Monthly	Weekly	Daily or almost daily
5. During the past year, how often have you failed to do what was normally expected of you because of drinking?	Never	Less than monthly	Monthly	Weekly	Daily or almost daily
6. During the past year, how often have you needed a drink in the morning to get yourself going after a heavy drinking session?	Never	Less than monthly	Monthly	Weekly	Daily or almost daily
7. During the past year, how often have you had a feeling of guilt or remorse after drinking?	Never	Less than monthly	Monthly	Weekly	Daily or almost daily
8. During the past year, have you been unable to remember what happened the night before because you had been drinking?	Never	Less than monthly	Monthly	Weekly	Daily or almost daily
9. Have you or someone else been injured as a result of your drinking?	No		Yes, but not in the past year		Yes, during the past year
10. Has a relative or friend, doctor or other health worker been concerned about your drinking or suggested you to cut down?	No		Yes, but not in the past year		Yes, during the past year

Note: If answered "Never" on first question, please proceed to questions 9 and 10.

12. How many times did you use the following substances? Please mark with "X" one box for each substance.

	Never Used	Once or Twice	3–5 Times	6–9 Times	10 Times or More
Ephedrine					
Cocaine					
Speed					
Ecstasy					
Cannabis/Marijuana					
Hashish					
LSD					
Heroin					
Ketamine					
GHB					
Sedatives (valium, vicodine, etc.)					
Inhalants (nitro, glue, etc.)					
